# Health in climate change research from 1990 to 2014: positive trend, but still underperforming

**DOI:** 10.3402/gha.v9.30723

**Published:** 2016-06-21

**Authors:** Glenn Verner, Stefanie Schütte, Juliane Knop, Osman Sankoh, Rainer Sauerborn

**Affiliations:** 1 Centre Virchow-Villermé for Public Health Paris-Berlin, Université Sorbonne Paris Cité, Paris, France; 2 French School of Public Health, Paris-Rennes, France; 3 INDEPTH Network, Accra, Ghana; 4 School of Public Health, Faculty of Health Sciences, University of the Witwatersrand, Johannesburg, South Africa; 5 Institute of Public Health, INF 324, University of Heidelberg, Heidelberg, Germany

**Keywords:** literature review, IPCC, NCDs, infectious diseases, malnutrition, respiratory diseases

## Abstract

**Background:**

Climate change has been recognized as both one of the biggest threats and the biggest opportunities for global health in the 21st century. This trend review seeks to assess and characterize the amount and type of scientific literature on the link between climate change and human health.

**Design:**

We tracked the use of climate-related terms and their co-occurrence with health terms during the 25 years since the first Intergovernmental Panel on Climate Change (IPCC) report, from 1990 to 2014, in two scientific databases and in the IPCC reports. We investigated the trends in the number of publications about health and climate change through time, by nature of the health impact under study, and by geographic area. We compared the scientific production in the health field with that of other sectors on which climate change has an impact.

**Results:**

The number of publications was extremely low in both databases from 1990 (325 and 1,004, respectively) until around 2006 (1,332 and 4,319, respectively), which has since then increased exponentially in recent years (6,079 and 17,395, respectively, in 2014). However, the number of climate change papers regarding health is still about half that of other sectors. Certain health impacts, particularly malnutrition and non-communicable diseases (NCDs), remain substantially understudied. Approximately two-thirds of all published studies were carried out in OECD countries (Organization for Economic Cooperation and Development), predominantly in Europe and North America.

**Conclusions:**

There is a clear need for further research on the links between climate change and health. This pertains particularly to research in and by those countries in which health will be mostly affected and capacity to adapt is least. Specific undertreated topics such as NCDs, malnutrition, and mental health should gain the priority they deserve. Funding agencies are invited to take note of and establish calls for proposals accordingly. Raising the interest in this research area in young scientists remains a challenge and should lead to innovative courses for large audiences, such as Massive Open Online Courses.

## Introduction

There is increasing international debate about climate change as the effects of fossil fuel emissions become more and more apparent. The 21st Conference of the Parties (COP21) in December 2015 attracted further interest in the topic. Climate change is a global concern that reaches across many sectors. Health effects resulting from climate change present us with both the greatest global threat and the ‘greatest global health opportunity of the 21st century’ (1), in that in combatting climate change, our actions can have direct benefits for public health (2). Although health could be an essential motivator for action on climate change, human health has often been neglected in climate change discourse in both research and dissemination (3–5).

Indeed in the early 1990s, there was very little awareness of the health risks caused by global climate change. The lack of scientific evidence on the public health effects of climate change was reflected in the content of the first assessment report – in 1990 – of the Intergovernmental Panel on Climate Change (IPCC), whose mandate is to assess the scientific evidence regarding climate change in the 5–7 years preceding each report (6). This initial report did not include a chapter on health and contained just a few scattered mentions of published papers. Subsequent reports included a health chapter, the size and scope of which has continued to grow through the recent 5th IPCC Assessment Report (AR) of 2014 (7, 8). One of the authors (RS) was a lead author of this report.

Particularly in the past decade, literature on the link between climate change and health has become more prominent. The Lancet Commission report on climate change and health in 2009 (9) counted more than 10,000 articles on this topic. In addition, several reviews of existing scientific literature on climate change and health have been published (10–12) that aim to summarize the evidence of the health impact due to climate change. In their scoping review, which seeks to identify research gaps in a topic too broad for a systematic review, Hosking and Campbell (13) focused exclusively on a narrow time frame of 2 years from January 2008 to June 2010 to compare the World Health Organization's research priorities published in 2008 (14) with subsequent publications in these areas during 30 months.

In the light of the recent Lancet Commission's call for further research on public health and climate change (1), we set out to seek answers on the following five questions through bibliometric searches:

1. What is the current volume of publications on climate change and health (henceforth abbreviated as CC&H)?
2. How does this compare with past trends since the very beginning of the field of CC&H, which we set at 1990; the Rio conference; and the signature of the UN Convention on Climate Change (15)?
3. What are the trends through time and current levels of research output by health impact, such as infectious diseases, malnutrition, and so on?
4. How does the amount of scientific publications compare with other sectors on which climate is known to have an important impact?
5. What is the geographical distribution of current scientific output on CC&H and how does this compare with the estimated magnitude of health impacts and vulnerability?

## Methods

We surveyed publications from 1990 to 2014, beginning in the year of the first IPCC report. The databases searched were PubMed and Science Direct and search terms were set and combined according to the database features (see Supplementary File). Science Direct was used because of its breadth and inclusion of topics outside of health, and PubMed was screened for its focus on medical and public health publications (16). The number of publications appearing were counted and plotted over time or across relevant categories. All searches were performed in English.

In order to be included for review, an article had to contain one climate term and one health term in either the title, abstract, or as a MeSH term (for PubMed searches). Climate change terms were selected following consultation of the literature and with experts to account for the differing terminology in use since 1990: climate change, global warming, climate variability, and greenhouse effect.

Overall, six different bibliometric searches were performed: 1 & 2) overall trend of publications on ‘climate change’ and ‘climate change and health’ (conducted in two separate databases); 3) number of publications on climate change and health in comparison with other sectors; 4) trends of publications according to health impact; 5) number of publications according to geographical region; and 6) number of mentions of ‘health’ in the IPCC reports (Table 1). Detailed search strategies are documented in Supplementary File.

**Table 1:** Six scoping searches on the topic of climate change and health with sources, key words used, and number of papers/mentions

Search	Topic	Source	Keywords^a^	No. of papers or occurrences
1	Climate change and health in the scientific literature	Science Direct	Health, disease, morbidity, mortality	44,193
2	Climate change and health in the scientific literature	PubMed	Health, disease, morbidity, mortality	5,237
3	Health and climate change versus other sectors	Science Direct	Transportation, industry, economy, energy	44,193
4	Health impacts of climate change	PubMed	Effects of extreme events, infectious disease, respiratory disease, nutrition, other effects	1,238
5	Regions studies	PubMed	World countries and regions (UN list)	1,187
6	‘Health’ term in IPCC reports	IPCC reports	Health	5,359

a Climate key words: ‘climate change’, ‘global warming’, ‘climate variability’, ‘greenhouse effect’.

In order to assess the overall trend of publications over time (search 1, search 2), a cluster of health-related search terms were used to capture a wide range of publications concerned with health issues (‘mortality’, ‘morbidity’, and ‘disease’). The number of publications including only a climate change key word (‘climate change’, ‘global warming’, ‘climate variability’, or ‘greenhouse effect’) was tracked in the same databases to show the proportion of climate change literature evoking health. The number of publications mentioning both climate change and health was compared with the number of publications linking climate change to four other important sectors (transportation, industry, economy, and energy) to provide context for our results (search 3). For search 4, we measured the number of publications linking climate change to specific health outcomes. The health impacts were classified according to the categories of climate change–related diseases given in the 5th IPCC Report into effects of extreme events, infectious disease, respiratory disease, nutrition, and others (7). Next, all captured publications were categorized according to the geographic region, using the United Nation lists of nations and regions as keywords in a database search (search 5) (17). The names of countries in their corresponding region (i.e. ‘Burkina Faso’ and ‘Western Africa’) were used as keywords to capture the maximum number of publications with minimum overlap and greater precision. The number of times ‘health’ is mentioned in the IPCC reports and where these references occur in the reports was counted as an indicator of the overall importance of health in the scientific discourse surrounding climate change and to provide further information on the treatment of climate change and health over time (search 6).

## Results

**Fig. 1:**
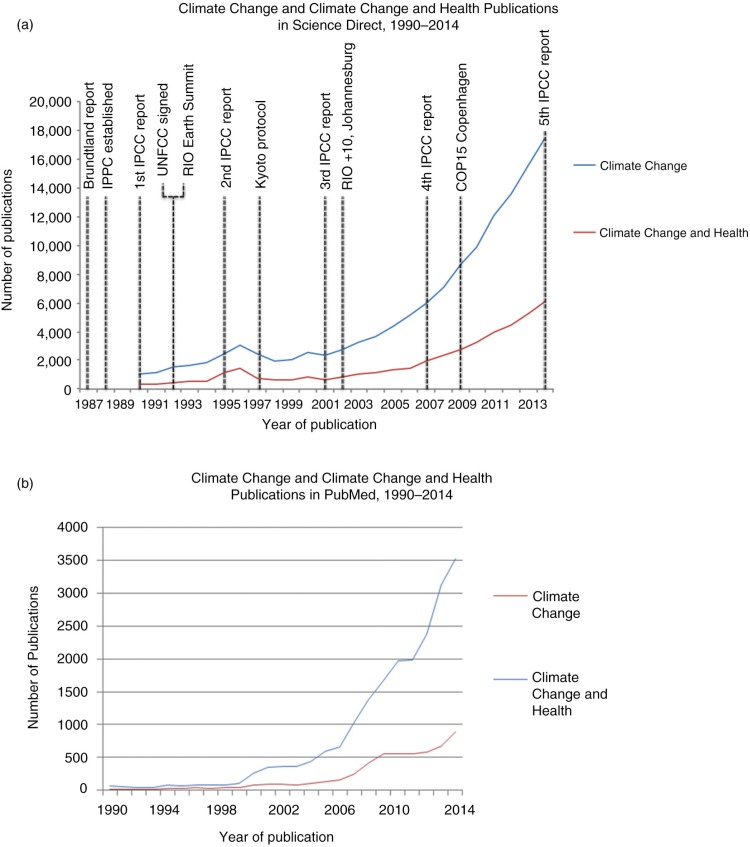
Publications on ‘climate change’ and ‘climate change and health’ by year indexed in Science Direct database (a) and PubMed database (b), 1990–2014. A search was performed to find articles on climate change mentioning both a climate change term (climate change, global warming, climate variability, or greenhouse effect) and a general health keyword (health, disease, morbidity, mortality). A second search was performed for publications containing only a climate change key word. Various important events and publications in the study of climate change are noted.

The number of publications between 1990 and 2014 on ‘climate change’ and ‘climate change and health’ are presented in Fig. 1a and b. Publications on health in Science Direct remained in the three-digit range until 2003 (barring a peak in 1996). We observed an exponential trend since 2003, albeit with a flatter slope for health publications (Fig. 1a). The search was also carried out in PubMed, where the same increasing trend was observed, with the number of publications on climate change and health crossing the three-digit mark in 2004, plateauing briefly in 2010, and continuing to increase through 2014 (Fig. 1b).

**Fig. 2:**
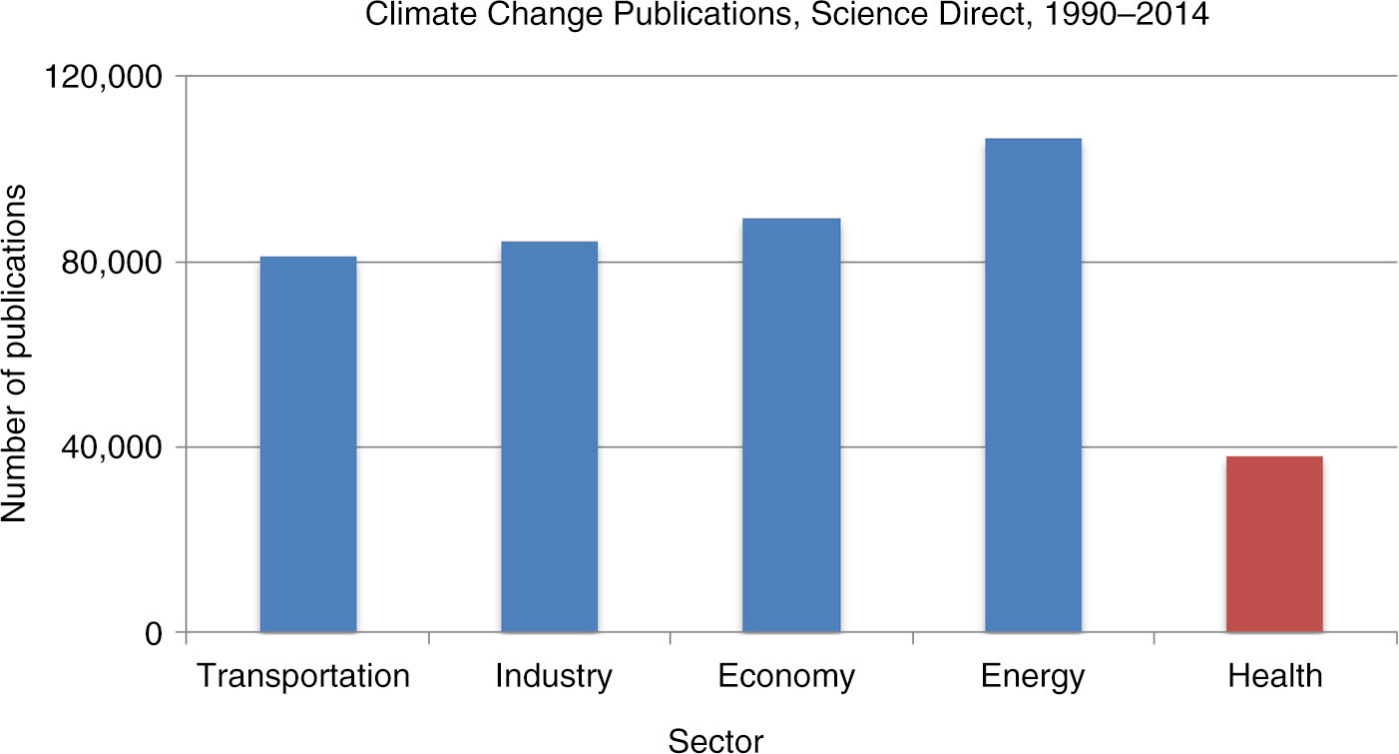
Comparison of articles mentioning climate change and other important sectors, which both emit and are affected by greenhouse gas emissions, Science Direct 1990–2014.

Figure 2 shows the number of publications per sector indexed in the database Science Direct (44, 193). Among these sectors, the topic of health in association with climate change was less studied. Transportation, industry, economy, and energy are each referenced in at least twice as many publications as the health sector.

**Fig. 3:**
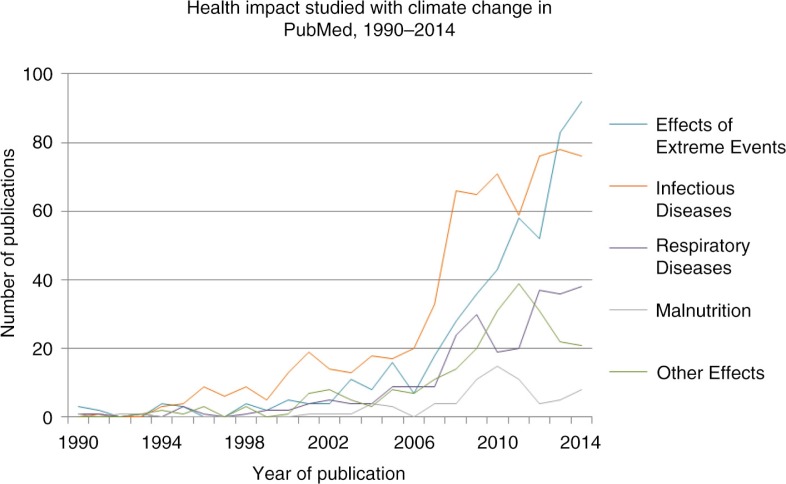
Climate change and health publications according to health impact studied in PubMed, 1990–2014. Categories of health impacts are taken from the 5th IPCC report, and the climate-related health outcomes given there are used as key words. Effects of extreme events result from extreme heat, flooding, and storms. Infectious diseases include vector-borne, water-borne, and food-borne diseases. Respiratory diseases encompass effects of air pollution, asthma, and allergies. Other effects of climate change on health include occupational health, mental health effects, migration, and conflict.

Figure 3 shows the different types of health impact explored in association with climate change. The number of publications in all health topic areas began to increase sharply in the early 2000s but show different trends in recent years.

Effects of extreme events (i.e. heat stroke, injuries, and cardiovascular disease) are the most studied, followed by infectious diseases, whereas there are few publications discussing both climate change and nutrition. Searching with general health terms (search 1) elicits more results than with specific health outcomes (search 2 – 3,999 fewer hits in PubMed).

**Fig. 4:**
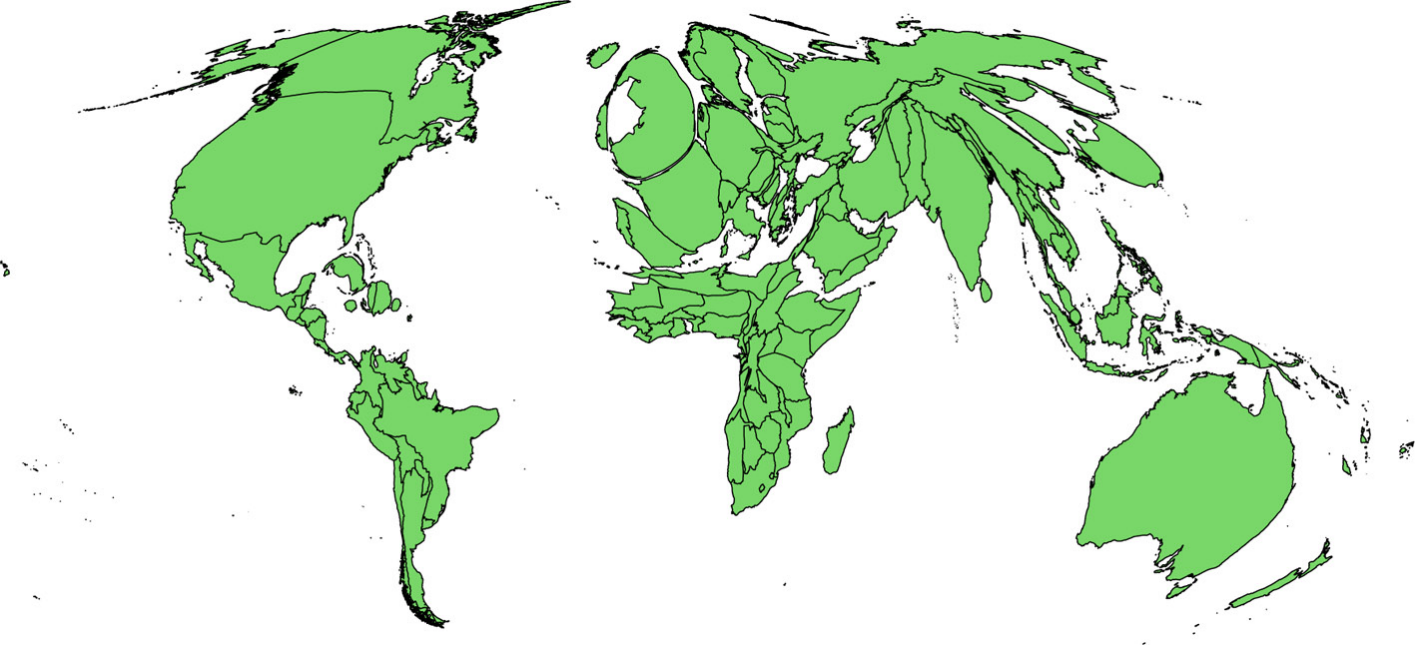
Publications on health and climate change by geographic region/country studied indexed in PubMed, 1990–2014. The depicted size of each country was altered to reflect the number of publications in those areas proportional to their population. Searches were performed using the UN list of nations and regions as key words (17). This cartogram was generated with the QGIS-cartogram plugin developed by Carson Farmer and Morten Wulff (18). The World Map Shapefile used to generate the cartogram was created by Bjorn Sandvik (19). Data from PubMed searches were provided for each UN subregion. As the shapefile and cartogram software depended on unique weights per country, the data were normalized against area for each country within each UN subregion. Data for the United Kingdom, the United States, and China were specifically isolated from their respective UN subregion and were provided with their own weights.

The publications on climate change and health by geographic region are presented in Fig. 4. There are strong geographic discrepancies in the regions studied. These numbers do not reflect the origin of the authors, which cannot be identified by a keyword search, but rather the country where the research was conducted. Approximately 66% of articles focus on the developed nations of Europe, North America, Australia, and New Zealand, while 7% mention Africa.

**Fig. 5:**
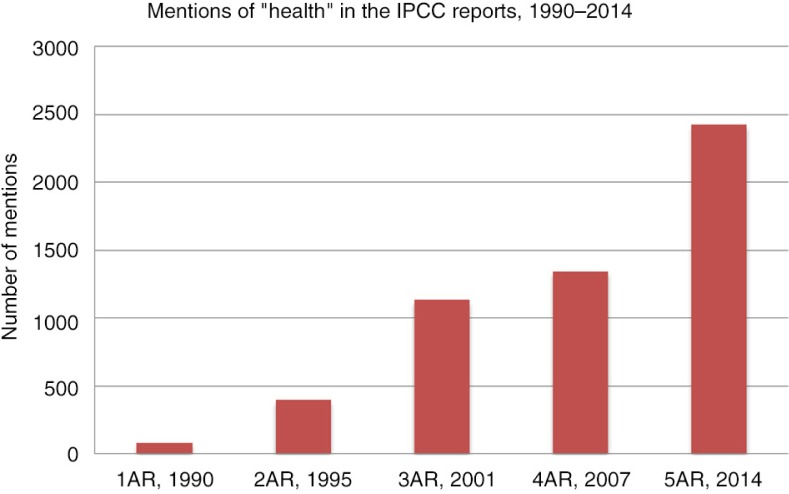
Number of times the word ‘health’ occurs in the IPCC reports, all chapters, 1990–2014.

As shown in Fig. 5, health has been increasingly mentioned in the IPCC reports, with the number of references doubling between AR2 and AR3 and again from AR4 to AR5. The number of pages in each health chapter also almost doubled between 1990 and 2014, and the number of pages in the executive summary referencing health increased from 19 (AR2) to 30 (AR5). Less than a third of the references in the most recent report (636 out of 2,418, 26%) are found within the health chapter, the rest occur throughout the report.

## Discussion

### Discussion of results

In the following section, we discuss to what extent we were able to answer the five questions posed in the introduction.

First, there is a positive and accelerating trend shown in publications on climate change and health. However, this trend is at a very low absolute level, and it would take years, if not decades, for the scientific community to close the gap if the productivity of research is the same as for other equally important risk factors for disease, such as smoking. These publications also continue to make up only a small minority of publications on climate change, and there remains much room for improvement in putting health and human effects in the center of the conversation around climate change. Health can be a motivating factor for action on climate change (20) and an increased focus on this in the scientific community could be a means of effecting individual and political change. Fresh ideas are needed, as universities do not sufficiently engage in formal courses raising the interest of young scientific minds in the topic of CC&H. Such courses may have on average 20 participants. We should explore other formats such as Massive Open Online Courses (MOOCs), which can attract thousands of participants, to reach out to larger audiences and combine them with traditional learning in innovative ways (21). For example, a MOOC on the basics of CC&H may raise the interest of 5% of participants who are then offered a presence course, a class where students and teachers are able to interact face to face, on research methods. A paper is in preparation analyzing the experience of our three MOOCs on climate change and health: one for policymakers/climate negotiators, one for the general public, and one by Africans for the African health context (5). Beyond a lack of available education, powerful political and commercial forces have incentives to downplay the health effects of climate change, raising further concern that future levels of publication will not rise to the level necessary to better inform both scientific research and government and individual action.

Second, the current number of publications that treat the subject of health related to climate change is still very low when compared with those of other climate-sensitive sectors. Hosking and Campbell-Lendrum (13) provide evidence that climate change as a risk factor for disease receives numbers of scientific publications which are orders of magnitude fewer than ‘established’ risk factors, such as smoking or obesity: within the decade from 2000 to 2010, the authors identified 875 citations on CC&H but only a mere 2% of the 47,862 papers on tobacco in the same period. Moreover, of the papers on CC&H, 42.7% were not original research but rather comments, editorials, letters, or reviews, compared with only 19.2% of the research on tobacco as a health risk factor. Sauerborn (22) proposed some reasons why many scientists from epidemiology or environmental health sciences do not pick up the topic of climate change. Discomfort with uncertainty which cannot be bound in statistical terms, such as p-values or confidence intervals, and the fact that effects in the future need to be modeled and are difficult to empirically assess given the long delays between exposure and outcomes are two proposed explanations.

Third, we observed a trend of ‘mainstreaming health’ in the debate on climate change impacts, reflecting an awareness of the diversity of possible health impacts. This is demonstrated in the IPCC reports, where in recent reports more mentions of health came from non-health chapters. The collaboration of other author teams with the health chapter team was clearly very active: participants from food security, vulnerability and adaptation, and agriculture groups, and the regional chapters such as on Africa and Asia all met and discussed overlapping topics and papers.

Fourth, apart from the increase in the absolute aggregate numbers of publications, we observed worrying gaps in the treatment of climate-sensitive diseases and conditions which, for example, the IPCC report considers of great importance. These topics include malnutrition, mental health, occupational health, and productivity limitations due to heat. Respiratory diseases including asthma, which are likely to be one of the largest health impacts from climate change, received about a third of the number of publications as on extreme events, largely heat waves (heat stroke, skin cancer, etc.), largely in northern cities.

Finally, we come to the issue of geographic disparities in publication output on CC&H. Figure 4 illustrates the gross mismatch between areas with high vulnerability to climate change on one side (i.e. sub-Saharan Africa) and those that are the focus of health research (i.e. the United Kingdom) on the other. One could argue that this reflects the lower number of researchers per population in Africa and much of Asia and demonstrates the general imbalance of scientific production. In fact, we did not attempt to compare whether the discrepancy between the size of health problems and the amount of research is larger in CC&H than in other areas, that is, cancer research (given that the vast majority of cancer patients live in poor countries, yet research originates predominately from rich ones). We can conclude, however, that this disparity between the impact of the health burden from climate change and the scientific effort allocated to it is unacceptable and needs to be remedied. Our finding of significant underrepresentation of Asia and Africa in the scientific production on CC&H dovetails well with the paper by Byass (23) and with the findings of Hosking and Campbell (13) on a smaller set of 80 studies included in their scoping review. Funding agencies and academic and non-academic institutions could partner with the many excellent research networks and centers in the global South in order to build research capacity where the problems are the greatest.

### Strengths and limitations of search methods

Our search methods yield an overall picture of the trends in climate change and health research from 1990 to 2014 (searches 1 and 2). In relying on database search engines to capture relevant articles, we risk including publications that would be filtered out in a systematic or scoping review. These searches will elicit results on all types of biological health, not only human health. Articles dealing with various health effects or regions may also have been included more than once. However, the trends in publications over time, health impact studied, and geographic region studied are so significant that it seems they would persist even taking into account a certain amount of misclassification, which in any case would not have been systematic. Moreover, our findings on the number of publications on climate change and health between 2008 and 2010 correspond with the results of Hosking and Campbell–Lendrum, providing confirmation for this method (13).

Both databases, PubMed and Science Direct, evidenced an increase in the number of publications on climate change and health, but to different degrees. In Science Direct, the number of publications on climate change and health increased in parallel with the publications on climate change alone. However, the number of publications on climate change and health articles in PubMed increased by a rate over 60% greater than publications on climate change alone. This is likely to be attributable to the difference in focus of the two databases. As Science Direct includes more articles on non-health-related topics, the relative proportion of climate change articles mentioning health would likely be less than in PubMed, which has a medical focus. Science Direct may therefore give a more accurate impression of the weight of the health argument among climate change scientists.

Search 3 used only specific health effects as key words (i.e. cardiovascular disease, malaria), not the general terms ‘health’, ‘disease’, ‘morbidity’, and ‘mortality’. The former searches produced far fewer results in total than the latter, although publications about specific health impacts ought to also be captured with the general health keywords (i.e. an article about malaria should also contain ‘health’, ‘disease’, ‘morbidity’, and/or ‘mortality’ as a key word(s)). This unexpected disparity between the number of results including specific health effects and the number of results about health writ large likely indicates that a majority of these publications do not contain original research and instead mention the health effects of climate change generally or transiently.

Our comparison of ‘climate change and health’ literature to climate change and other sectors was carried out in Science Direct due to its broader scope. These sectors were chosen because, like health, they both emit and are impacted by climate change to a greater or lesser degree. We demonstrate that health is evoked less than other themes across climate change literature as a whole. Our method does not preclude the same publication containing several of the selected terms and therefore being counted multiple times. Nevertheless, the level of difference of treatment of health is so large that it can be expected to persist even taking this into account.

Searches by region were carried out using the names of each country as key words, grouped into geographic areas according to the United Nations regional list (17). Although this method may not capture some articles, which use regional or natural references to refer to their area of study (i.e. sub-Saharan, Arctic, coastal), it is more exhaustive than other groupings. The majority of authors include the country (countries) that the article studies, and there should be no systematic bias distinguishing those who do not. We find another discrepancy in the number of results obtained with this method and the number of results found with general health keywords, again implying that articles on climate change and health lack grounding in a specific context and do not present original data.

We observed a marked increase in the number of references to ‘health’ between the first and the fifth IPCC reports and a greater number of pages containing ‘health’ across the entirety of the reports (7). This increase may not only be due to greater focus given to health in climate change research alone but also to stylistic choices, increased linking of sections within the report, or a wider range of literature reviewed. Nevertheless, we can see an expansion of the role of health in the statements of the IPCC, mirroring an increased treatment of the topic in the literature.

## Summary

This review noted an increase in publications on climate change and health between 1990 and 2014 in the literature from two scientific databases, PubMed and Science Direct, as well as in the IPCC reports. In addition, our results demonstrated a continuing underrepresentation of certain topics, health effects, and regions where climate change is projected to have important effects. In spite of the increasing focus on the public health implications of a changing climate on the part of civil society and governments and multilateral bodies, publications on health continue to constitute only a minority of the literature on climate change.

## Conclusion

The problems of scientific underproduction and mismatch between the research topics and the nature of health impacts and the locations where people will suffer most from climate change will not go away, unless a concerted effort is orchestrated bringing together research funding agencies, philanthropists, universities, and non-academic scientific institutions from north and south to address this problem (19). Funding alone is not the solution. Research interest needs to be sparked, research capacity needs to be increased considerably, and public health researchers need to be taught as to how to use climate models and cooperate with scientists from wide-ranging disciplines, namely meteorology, climate modeling, remote sensing, agriculture, policy, and more. This buildup of research capacity must take place in both the global North and the South, but with particular emphasis in the South, to make health an important part of action on climate change.

## Supplementary Material

Health in climate change research from 1990 to 2014: positive trend, but still underperforming

## Paper Context

Climate change has become an increasingly important topic in scientific dialogue, political institutions, and the media. Human health will be fundamentally affected by climate change and can serve as a unifying motivator for action, but has been undertreated in the climate change discourse. Our review seeks to characterize the amount and type of scientific publications on climate change and health since the publication of the first IPCC report in 1990.
