# Supplementary material for: Health in climate change research from 1990 to 2014: positive trend, but still underperforming
Source: Glob Health Action. 2016 Jun 21;9:10.3402/gha.v9.30723. doi: 10.3402/gha.v9.30723 (PMC4917601; doi:10.3402/gha.v9.30723)
Supplement: Health in climate change research from 1990 to 2014: positive trend, but still underperforming [file GHA-9-30723-s001.pdf]

Supplementary material:

Health in climate change research from 1990 to 2014: positive trend, but still underperforming

Global Health Action 2016; 8:30723

Search terms

Climate change:

PubMed: ("climate change"[All Fields] OR "global warming"[All Fields] OR "climate variability"[All Fields] OR "greenhouse effect"[All Fields]) AND ("Health"[Mesh] OR "Disease"[Mesh] OR "Morbidity"[Mesh] OR "epidemiology"[Subheading] OR "Mortality"[Mesh] OR "mortality"[Subheading]) AND ("1990/01/01"[PDAT] : "2014/12/31"[PDAT])

ScienceDirect: ("climate change" OR "global warming" OR "climate variability" OR "greenhouse effect"), date range set 1990-2014

Climate change and health:

PubMed: ("climate change"[All Fields] OR "global warming"[All Fields] OR "climate variability"[All Fields] OR "greenhouse effect"[All Fields]) AND ("Health"[Mesh] OR "Disease"[Mesh] OR "Morbidity"[Mesh] OR "epidemiology"[Subheading] OR "Mortality"[Mesh] OR "mortality"[Subheading]) AND ("1990/01/01"[PDAT] : "2014/12/31"[PDAT])

ScienceDirect: ("climate change" OR "global warming" OR "climate variability OR "greenhouse effect”) AND ("Health” OR "Disease" OR "Morbidity" OR "Mortality" OR "mortality"), date range set 1990-2014

Climate change and transportation:

ScienceDirect: ("climate change" OR "global warming" OR "climate variability" OR "greenhouse effect") AND transport*

Climate change and industry:

ScienceDirect: ("climate change" OR "global warming" OR "climate variability" OR "greenhouse effect") AND industr*

Climate change and economy:

ScienceDirect: ("climate change" OR "global warming" OR "climate variability" OR "greenhouse effect") AND econom*

Climate change and energy:

ScienceDirect: ("climate change" OR "global warming" OR "climate variability" OR "greenhouse effect") AND energ*

Climate change and health:

ScienceDirect: ("climate change" OR "global warming" OR "climate variability" OR "greenhouse effect") AND health

Climate change and health, direct effects:

PubMed: ("climate change"[All Fields] OR "global warming"[All Fields] OR "climate variability"[All Fields] OR "greenhouse effect"[All Fields]) AND ("Heat Exhaustion"[Mesh] OR "Heat Stress Disorders"[Mesh] OR "Heat Stroke"[Mesh] OR "Sunstroke"[Mesh] OR "Heat-Shock Response"[Mesh] OR "Hypothermia"[Mesh] OR "Shock"[Mesh] OR "Cardiovascular Diseases"[Mesh] OR "Kidney Diseases"[Mesh] OR "Wounds and Injuries"[Mesh] OR "Drowning"[Mesh] OR "Skin Neoplasms"[Mesh] OR "Cataract"[Mesh] OR "Dehydration"[Mesh]) AND ("1990/01/01"[PDAT] : "2014/12/31"[PDAT])

Climate change and health, infectious disease:

PubMed: ("climate change"[All Fields] OR "global warming"[All Fields] OR "climate variability"[All Fields] OR "greenhouse effect"[All Fields]) AND ("Malaria"[Mesh] OR "Dengue"[Mesh] OR "Encephalitis, Tick-Borne"[Mesh] OR "Tick-Borne Diseases"[Mesh] OR "Chikungunya Fever"[Mesh] OR "West Nile Fever"[Mesh] OR "Rift Valley fever virus"[Mesh] OR "Encephalitis, Japanese"[Mesh] OR "Hemorrhagic Fever with Renal Syndrome"[Mesh] OR "Hantaan virus"[Mesh] OR "Plague"[Mesh] OR "Lyme Disease"[Mesh] OR "Vibrio"[Mesh] OR "Cholera"[Mesh] OR "Salmonella"[Mesh] OR "Campylobacter"[Mesh] OR "Diarrhea"[Mesh] OR "Salmonella Infections"[Mesh] OR "Campylobacter Infections"[Mesh] OR "Enterovirus Infections"[Mesh] OR "Enterovirus"[Mesh] OR "Coxsackievirus Infections"[Mesh] OR "Hand, Foot and Mouth Disease"[Mesh] OR "Rotavirus"[Mesh] OR "Rotavirus Infections"[Mesh] OR "Harmful Algal Bloom"[Mesh] OR "Dysentery"[Mesh] OR "Schistosomiasis"[Mesh]) AND ("1990/01/01"[PDAT] : "2014/12/31"[PDAT])

Climate change and health, respiratory disease:

PubMed: ("climate change"[All Fields] OR "global warming"[All Fields] OR "climate variability"[All Fields] OR "greenhouse effect"[All Fields]) AND ("Respiratory Tract Diseases"[Mesh] OR "Respiration Disorders"[Mesh] OR "Hypersensitivity"[Mesh] OR "Allergy and Immunology"[Mesh] OR "Rhinitis, Allergic"[Mesh] OR "Rhinitis, Allergic, Seasonal"[Mesh] OR "Conjunctivitis, Allergic"[Mesh] OR "Dermatitis"[Mesh] OR "Eczema"[Mesh]) AND ("1990/01/01"[PDAT] : "2014/12/31"[PDAT])

Climate change and health, nutrition:

PubMed: ("climate change"[All Fields] OR "global warming"[All Fields] OR "climate variability"[All Fields] OR "greenhouse effect"[All Fields]) AND ("Nutritional Status"[Mesh] OR "Fetal Nutrition Disorders"[Mesh] OR "Child Nutrition Disorders"[Mesh] OR "Infant Nutrition Disorders"[Mesh] OR "Malnutrition"[Mesh] OR "Wasting Syndrome"[Mesh] OR "Thinness"[Mesh] OR "Food Supply"[Mesh]) AND ("1990/01/01"[PDAT] : "2014/12/31"[PDAT])

Climate change and health, human system mediated:

PubMed: ("climate change"[All Fields] OR "global warming"[All Fields] OR "climate variability"[All Fields] OR "greenhouse effect"[All Fields]) AND ("Occupational Health"[Mesh] OR "Mental

Health"[Mesh] OR "Mental Disorders"[Mesh] OR "Stress Disorders, Post-Traumatic"[Mesh] OR "Stress Disorders, Traumatic"[Mesh] OR "Anxiety Disorders"[Mesh] OR "Depression"[Mesh] OR "Depressive Disorder"[Mesh] OR "Aggression"[Mesh] OR "Suicide"[Mesh] OR "Suicide, Attempted"[Mesh] OR "Violence"[Mesh] OR "Human Migration"[Mesh] OR "Emigration and Immigration"[Mesh] OR "Refugees"[Mesh] OR "Ethnic Conflict"[Mesh] OR "War"[Mesh]) AND ("1990/01/01"[PDAT] : "2014/12/31"[PDAT]) AND ("1990/01/01"[PDAT] : "2014/12/31"[PDAT])

Climate change and health, Eastern Africa:

PubMed: ("climate change"[All Fields] OR "global warming"[All Fields] OR "climate variability"[All Fields] OR "greenhouse effect"[All Fields]) AND ("Health"[Mesh] OR "Disease"[Mesh] OR "Morbidity"[Mesh] OR "epidemiology"[Subheading] OR "Mortality"[Mesh] OR "mortality"[Subheading]) AND ("Africa, Eastern"[Mesh] OR "Burundi"[Mesh] OR "Comoros"[Mesh] OR "Djibouti"[Mesh] OR "Eritrea"[Mesh] OR "Ethiopia"[Mesh] OR "Kenya"[Mesh] OR "Madagascar"[Mesh] OR "Malawi"[Mesh] OR "Mauritius"[Mesh] OR "Mozambique"[Mesh] OR "Reunion"[Mesh] OR "Rwanda"[Mesh] OR "Seychelles"[Mesh] OR "Uganda"[Mesh] OR "Tanzania"[Mesh] OR "Zambia"[Mesh] OR "Zimbabwe"[Mesh]) AND ("1990/01/01"[PDAT] : "2014/12/31"[PDAT])

Climate change and health, Middle Africa:

PubMed: ("climate change"[All Fields] OR "global warming"[All Fields] OR "climate variability"[All Fields] OR "greenhouse effect"[All Fields]) AND ("Health"[Mesh] OR "Disease"[Mesh] OR "Morbidity"[Mesh] OR "epidemiology"[Subheading] OR "Mortality"[Mesh] OR "mortality"[Subheading]) AND ("Angola"[Mesh] OR "Cameroon"[Mesh] OR "Central African Republic"[Mesh] OR "Africa, Central"[Mesh] OR "Chad"[Mesh] OR "Congo"[Mesh] OR "Democratic Republic of the Congo"[Mesh] OR "Equatorial Guinea"[Mesh] OR "Gabon"[Mesh]) AND ("1990/01/01"[PDAT] : "2915/12/31"[PDAT])

Climate change and health, Northern Africa:

PubMed: ("climate change"[All Fields] OR "global warming"[All Fields] OR "climate variability"[All Fields] OR "greenhouse effect"[All Fields]) AND ("Health"[Mesh] OR "Disease"[Mesh] OR "Morbidity"[Mesh] OR "epidemiology"[Subheading] OR "Mortality"[Mesh] OR "mortality"[Subheading]) AND ("Africa, Northern"[Mesh] OR "Algeria"[Mesh] OR "Egypt"[Mesh] OR "Libya"[Mesh] OR "Morocco"[Mesh] OR "Sudan"[Mesh] OR "Tunisia"[Mesh]) AND ("1990/01/01"[PDAT] : "2014/12/31"[PDAT])

Climate change and health, Southern Africa:

PubMed: ("climate change"[All Fields] OR "global warming"[All Fields] OR "climate variability"[All Fields] OR "greenhouse effect"[All Fields]) AND ("Health"[Mesh] OR "Disease"[Mesh] OR "Morbidity"[Mesh] OR "epidemiology"[Subheading] OR "Mortality"[Mesh] OR "mortality"[Subheading]) AND ("Africa, Southern"[Mesh] OR "Botswana"[Mesh] OR "Lesotho"[Mesh] OR "Namibia"[Mesh] OR "South Africa"[Mesh] OR "Swaziland"[Mesh]) AND ("1990/01/01"[PDAT] : "2014/12/31"[PDAT])

Climate change and health, Western Africa:

PubMed: ("climate change"[All Fields] OR "global warming"[All Fields] OR "climate variability"[All Fields] OR "greenhouse effect"[All Fields]) AND ("Health"[Mesh] OR "Disease"[Mesh] OR "Morbidity"[Mesh] OR "epidemiology"[Subheading] OR "Mortality"[Mesh] OR "mortality"[Subheading]) AND ("Africa, Western"[Mesh] OR "Benin"[Mesh] OR "Burkina Faso"[Mesh] OR "Cape Verde"[Mesh] OR "Gambia"[Mesh] OR "Ghana"[Mesh] OR "Guinea"[Mesh] OR "Guinea-Bissau"[Mesh] OR "Liberia"[Mesh] OR "Mali"[Mesh] OR "Mauritania"[Mesh] OR "Niger"[Mesh] OR "Senegal"[Mesh] OR "Sierra Leone"[Mesh] OR "Togo"[Mesh]) AND ("1990/01/01"[PDAT] : "2915/12/31"[PDAT])

Climate change and health, Caribbean:

PubMed: ("climate change"[All Fields] OR "global warming"[All Fields] OR "climate variability"[All Fields] OR "greenhouse effect"[All Fields]) AND ("Health"[Mesh] OR "Disease"[Mesh] OR "Morbidity"[Mesh] OR "epidemiology"[Subheading] OR "Mortality"[Mesh] OR "mortality"[Subheading]) AND ("Caribbean Region"[Mesh] OR "West Indies"[Mesh] OR "Anguilla"[Mesh] OR "Antigua and Barbuda"[Mesh] OR "Bahamas"[Mesh] OR "Barbados"[Mesh] OR "Netherlands Antilles"[Mesh] OR "British Virgin Islands"[Mesh] OR "Cuba"[Mesh] OR "Dominica"[Mesh] OR "Dominican Republic"[Mesh] OR "Grenada"[Mesh] OR "Guadeloupe"[Mesh] OR "Haiti"[Mesh] OR "Jamaica"[Mesh] OR "Martinique"[Mesh] OR "Puerto Rico"[Mesh] OR "Saint Kitts and Nevis"[Mesh] OR "Saint Lucia"[Mesh] OR "Saint Vincent and the Grenadines"[Mesh] OR "Trinidad and Tobago"[Mesh] OR "United States Virgin Islands"[Mesh]) AND ("1990/01/01"[PDAT] : "2014/12/31"[PDAT])

Climate change and health, Central America:

PubMed: ("climate change"[All Fields] OR "global warming"[All Fields] OR "climate variability"[All Fields] OR "greenhouse effect"[All Fields]) AND ("Health"[Mesh] OR "Disease"[Mesh] OR "Morbidity"[Mesh] OR "epidemiology"[Subheading] OR "Mortality"[Mesh] OR "mortality"[Subheading]) AND ("Central America"[Mesh] OR "Belize"[Mesh] OR "Costa Rica"[Mesh] OR "El Salvador"[Mesh] OR "Guatemala"[Mesh] OR "Honduras"[Mesh] OR "Mexico"[Mesh] OR "Nicaragua"[Mesh] OR "Panama"[Mesh]) AND ("1990/01/01"[PDAT] : "2014/12/31"[PDAT])

Climate change and health, South America:

PubMed: ("climate change"[All Fields] OR "global warming"[All Fields] OR "climate variability"[All Fields] OR "greenhouse effect"[All Fields]) AND ("Health"[Mesh] OR "Disease"[Mesh] OR "Morbidity"[Mesh] OR "epidemiology"[Subheading] OR "Mortality"[Mesh] OR "mortality"[Subheading]) AND ("South America"[Mesh] OR "Argentina"[Mesh] OR "Bolivia"[Mesh] OR "Brazil"[Mesh] OR "Chile"[Mesh] OR "Colombia"[Mesh] OR "Ecuador"[Mesh] OR "Falkland Islands"[Mesh] OR "French Guiana"[Mesh] OR "Guyana"[Mesh] OR "Paraguay"[Mesh] OR "Peru"[Mesh] OR "Suriname"[Mesh] OR "Uruguay"[Mesh] OR "Venezuela"[Mesh]) AND ("1990/01/01"[PDAT] : "2014/12/31"[PDAT])

Climate change and health, North America:

PubMed: ("climate change"[All Fields] OR "global warming"[All Fields] OR "climate variability"[All Fields] OR "greenhouse effect"[All Fields]) AND ("Health"[Mesh] OR "Disease"[Mesh] OR "Morbidity"[Mesh] OR "epidemiology"[Subheading] OR "Mortality"[Mesh] OR

"mortality"[Subheading]) AND ("North America"[Mesh] OR "Bermuda"[Mesh] OR "Canada"[Mesh] OR "Greenland"[Mesh] OR "United States"[Mesh]) AND ("1990/01/01"[PDAT] : "2014/12/31"[PDAT])

Climate change and health, Central Asia:

PubMed: ("climate change"[All Fields] OR "global warming"[All Fields] OR "climate variability"[All Fields] OR "greenhouse effect"[All Fields]) AND ("Health"[Mesh] OR "Disease"[Mesh] OR "Morbidity"[Mesh] OR "epidemiology"[Subheading] OR "Mortality"[Mesh] OR "mortality"[Subheading]) AND ("Asia, Central"[Mesh] OR "Kazakhstan"[Mesh] OR "Kyrgyzstan"[Mesh] OR "Tajikistan"[Mesh] OR "Turkmenistan"[Mesh] OR "Uzbekistan"[Mesh]) AND ("1990/01/01"[PDAT] : "2014/12/31"[PDAT])

Climate change and health, Eastern Asia:

PubMed: ("climate change"[All Fields] OR "global warming"[All Fields] OR "climate variability"[All Fields] OR "greenhouse effect"[All Fields]) AND ("Health"[Mesh] OR "Disease"[Mesh] OR "Morbidity"[Mesh] OR "epidemiology"[Subheading] OR "Mortality"[Mesh] OR "mortality"[Subheading]) AND ("Far East"[Mesh] OR "China"[Mesh] OR "Hong Kong"[Mesh] OR "Macau"[Mesh] OR "Japan"[Mesh] OR "Democratic People's Republic of Korea"[Mesh] OR "Republic of Korea"[Mesh] OR "Korea"[Mesh] OR "Mongolia"[Mesh] OR "Taiwan"[Mesh]) AND ("1990/01/01"[PDAT] : "2014/12/31"[PDAT])

Climate change and health, Southern Asia:

PubMed: ("climate change"[All Fields] OR "global warming"[All Fields] OR "climate variability"[All Fields] OR "greenhouse effect"[All Fields]) AND ("Health"[Mesh] OR "Disease"[Mesh] OR "Morbidity"[Mesh] OR "epidemiology"[Subheading] OR "Mortality"[Mesh] OR "mortality"[Subheading]) AND ("Asia, Western"[Mesh] OR "Afghanistan"[Mesh] OR "Bangladesh"[Mesh] OR "Bhutan"[Mesh] OR "India"[Mesh] OR "Iran"[Mesh] OR "Nepal"[Mesh] OR "Pakistan"[Mesh] OR "Sri Lanka"[Mesh]) AND ("1990/01/01"[PDAT] : "2014/12/31"[PDAT])

Climate change and health, South-Eastern Asia:

PubMed: ("climate change"[All Fields] OR "global warming"[All Fields] OR "climate variability"[All Fields] OR "greenhouse effect"[All Fields]) AND ("Health"[Mesh] OR "Disease"[Mesh] OR "Morbidity"[Mesh] OR "epidemiology"[Subheading] OR "Mortality"[Mesh] OR "mortality"[Subheading]) AND ("Asia, Southeastern"[Mesh] OR "Brunei"[Mesh] OR "Cambodia"[Mesh] OR "Indonesia"[Mesh] OR "Malaysia"[Mesh] OR "Myanmar"[Mesh] OR "Philippines"[Mesh] OR "Singapore"[Mesh] OR "Thailand"[Mesh] OR "East Timor"[Mesh] OR "Vietnam"[Mesh]) AND ("1990/01/01"[PDAT] : "2014/12/31"[PDAT])

Climate change and health, Western Asia:

PubMed: ("climate change"[All Fields] OR "global warming"[All Fields] OR "climate variability"[All Fields] OR "greenhouse effect"[All Fields]) AND ("Health"[Mesh] OR "Disease"[Mesh] OR "Morbidity"[Mesh] OR "epidemiology"[Subheading] OR "Mortality"[Mesh] OR "mortality"[Subheading]) AND ("Middle East"[Mesh] OR "Armenia"[Mesh] OR "Azerbaijan"[Mesh] OR "Bahrain"[Mesh] OR "Cyprus"[Mesh] OR "Georgia (Republic)"[Mesh] OR "Iraq"[Mesh] OR

"Israel"[Mesh] OR "Jordan"[Mesh] OR "Kuwait"[Mesh] OR "Lebanon"[Mesh] OR "Oman"[Mesh] OR "Qatar"[Mesh] OR "Saudi Arabia"[Mesh] OR "Syria"[Mesh] OR "Turkey"[Mesh] OR "United Arab Emirates"[Mesh] OR "Yemen"[Mesh]) AND ("1990/01/01"[PDAT] : "2014/12/31"[PDAT])

Climate change and health, Eastern Europe:

PubMed: ("climate change"[All Fields] OR "global warming"[All Fields] OR "climate variability"[All Fields] OR "greenhouse effect"[All Fields]) AND ("Health"[Mesh] OR "Disease"[Mesh] OR "Morbidity"[Mesh] OR "epidemiology"[Subheading] OR "Mortality"[Mesh] OR "mortality"[Subheading]) AND ("Europe, Eastern"[Mesh] OR "Republic of Belarus"[Mesh] OR "Bulgaria"[Mesh] OR "Czech Republic"[Mesh] OR "Hungary"[Mesh] OR "Poland"[Mesh] OR "Moldova"[Mesh] OR "Romania"[Mesh] OR "Russia"[Mesh] OR "Slovakia"[Mesh] OR "Ukraine"[Mesh]) AND ("1990/01/01"[PDAT] : "2014/12/31"[PDAT])

Climate change and health, Northern Europe:

PubMed: ("climate change"[All Fields] OR "global warming"[All Fields] OR "climate variability"[All Fields] OR "greenhouse effect"[All Fields]) AND ("Health"[Mesh] OR "Disease"[Mesh] OR "Morbidity"[Mesh] OR "epidemiology"[Subheading] OR "Mortality"[Mesh] OR "mortality"[Subheading]) AND ("Channel Islands"[Mesh] OR "Denmark"[Mesh] OR "Estonia"[Mesh] OR "Finland"[Mesh] OR "Guernsey"[Mesh] OR "Iceland"[Mesh] OR "Ireland"[Mesh] OR "Latvia"[Mesh] OR "Lithuania"[Mesh] OR "Norway"[Mesh] OR "Sweden"[Mesh] OR "Great Britain"[Mesh] OR "Northern Ireland"[Mesh]) AND ("1990/01/01"[PDAT] : "2014/12/31"[PDAT])

Climate change and health, Southern Europe:

PubMed: ("climate change"[All Fields] OR "global warming"[All Fields] OR "climate variability"[All Fields] OR "greenhouse effect"[All Fields]) AND ("Health"[Mesh] OR "Disease"[Mesh] OR "Morbidity"[Mesh] OR "epidemiology"[Subheading] OR "Mortality"[Mesh] OR "mortality"[Subheading]) AND ("Albania"[Mesh] OR "Andorra"[Mesh] OR "Bosnia-Herzegovina"[Mesh] OR "Croatia"[Mesh] OR "Gibraltar"[Mesh] OR "Greece"[Mesh] OR "Vatican City"[Mesh] OR "Italy"[Mesh] OR "Malta"[Mesh] OR "Montenegro"[Mesh] OR "Portugal"[Mesh] OR "San Marino"[Mesh] OR "Serbia"[Mesh] OR "Slovenia"[Mesh] OR "Spain"[Mesh] OR "Macedonia (Republic)"[Mesh]) AND ("1990/01/01"[PDAT] : "2014/12/31"[PDAT])

Climate change and health, Western Europe:

PubMed: ("climate change"[All Fields] OR "global warming"[All Fields] OR "climate variability"[All Fields] OR "greenhouse effect"[All Fields]) AND ("Health"[Mesh] OR "Disease"[Mesh] OR "Morbidity"[Mesh] OR "epidemiology"[Subheading] OR "Mortality"[Mesh] OR "mortality"[Subheading]) AND ("Austria"[Mesh] OR "Belgium"[Mesh] OR "France"[Mesh] OR "Germany"[Mesh] OR "Liechtenstein"[Mesh] OR "Luxembourg"[Mesh] OR "Monaco"[Mesh] OR "Netherlands"[Mesh] OR "Switzerland"[Mesh]) AND ("1990/01/01"[PDAT] : "2014/12/31"[PDAT])

Climate change and health, Australia and New Zealand:

PubMed: ("climate change"[All Fields] OR "global warming"[All Fields] OR "climate variability"[All Fields] OR "greenhouse effect"[All Fields]) AND ("Health"[Mesh] OR "Disease"[Mesh] OR

"Morbidity"[Mesh] OR "epidemiology"[Subheading] OR "Mortality"[Mesh] OR "mortality"[Subheading]) AND ("Australia"[Mesh] OR "New Zealand"[Mesh]) AND ("1990/01/01"[PDAT] : "2014/12/31"[PDAT])

Climate change and health, Melanesia:

PubMed: ("climate change"[All Fields] OR "global warming"[All Fields] OR "climate variability"[All Fields] OR "greenhouse effect"[All Fields]) AND ("Health"[Mesh] OR "Disease"[Mesh] OR "Morbidity"[Mesh] OR "epidemiology"[Subheading] OR "Mortality"[Mesh] OR "mortality"[Subheading]) AND ("Melanesia"[Mesh] OR "Fiji"[Mesh] OR "New Caledonia"[Mesh] OR "Papua New Guinea"[Mesh] OR "Vanuatu"[Mesh]) AND ("1990/01/01"[PDAT] : "2014/12/31"[PDAT])

Climate change and health, Micronesia:

PubMed: ("climate change"[All Fields] OR "global warming"[All Fields] OR "climate variability"[All Fields] OR "greenhouse effect"[All Fields]) AND ("Health"[Mesh] OR "Disease"[Mesh] OR "Morbidity"[Mesh] OR "epidemiology"[Subheading] OR "Mortality"[Mesh] OR "mortality"[Subheading]) AND ("Micronesia"[Mesh] OR "Guam"[Mesh] OR "Palau"[Mesh]) AND ("1990/01/01"[PDAT] : "2014/12/31"[PDAT])

Climate change and health, Polynesia:

PubMed: ("climate change"[All Fields] OR "global warming"[All Fields] OR "climate variability"[All Fields] OR "greenhouse effect"[All Fields]) AND ("Health"[Mesh] OR "Disease"[Mesh] OR "Morbidity"[Mesh] OR "epidemiology"[Subheading] OR "Mortality"[Mesh] OR "mortality"[Subheading]) AND ("Polynesia"[Mesh] OR "American Samoa"[Mesh] OR "Pitcairn Island"[Mesh] OR "Samoa"[Mesh] OR "Tonga"[Mesh]) AND ("1990/01/01"[PDAT] : "2014/12/31"[PDAT])
